# A DNA packaging motor inchworms along one strand allowing it to adapt to alternative double-helical structures

**DOI:** 10.1038/s41467-021-23725-5

**Published:** 2021-06-08

**Authors:** Juan P. Castillo, Alexander B. Tong, Sara Tafoya, Paul J. Jardine, Carlos Bustamante

**Affiliations:** 1 Jason L. Choy Laboratory of Single-Molecule Biophysics, University of California, Berkeley, CA USA; 2 Institute for Quantitative Biosciences-QB3, University of California, Berkeley, Berkeley, CA USA; 3 Chemistry Graduate Group, University of California, Berkeley, Berkeley, CA USA; 4 Biophysics Graduate Group, University of California, Berkeley, Berkeley, CA USA; 5 Department of Diagnostic and Biological Sciences and Institute for Molecular Virology, University of Minnesota, Minneapolis, MN USA; 6 Department of Molecular and Cell Biology, University of California, Berkeley, Berkeley, CA USA; 7 Department of Chemistry, University of California, Berkeley, Berkeley, CA USA; 8 Department of Physics, University of California, Berkeley, Berkeley, CA USA; 9 Howard Hughes Medical Institute, University of California, Berkeley, Berkeley, CA USA; 10 Kavli Energy Nanoscience Institute, University of California, Berkeley, Berkeley, CA USA; 11 Present Address: SMAT-C, Departamento de Física, Facultad de Ciencia, Universidad de Santiago, Santiago, Chile

**Keywords:** Single-molecule biophysics, Molecular biology

## Abstract

Ring ATPases that translocate disordered polymers possess lock-washer architectures that they impose on their substrates during transport via a *hand-over-hand* mechanism. Here, we investigate the operation of ring motors that transport ordered, helical substrates, such as the bacteriophage ϕ29 dsDNA packaging motor. This pentameric motor alternates between an ATP loading *dwell* and a hydrolysis *burst* wherein it packages one turn of DNA in four steps. When challenged with DNA-RNA hybrids and dsRNA, the motor matches its burst to the shorter helical pitches, keeping three power strokes invariant while shortening the fourth. Intermittently, the motor loses grip on the RNA-containing substrates, indicating that it makes optimal load-bearing contacts with dsDNA. To rationalize these observations, we propose a *helical inchworm* translocation mechanism in which, during each cycle, the motor increasingly adopts a lock-washer structure during the ATP loading dwell and successively regains its planar form with each power stroke during the burst.

## Abstract (web summary)

Ring ATPase translocases that operate on disordered substrates adopt lockwasher architectures and use a hand-over-hand mechanism. By challenging the dsDNA packaging motor of bacteriophage ϕ29 with hybrid and dsRNA, the authors propose that the motor cycles between planar and lock-washer structures.

## Introduction

Ring-shaped ATPases of the ASCE division convert the energy of nucleotide hydrolysis into mechanical work to drive a variety of processes inside living cells, from electro-chemical gradient buildup to protein degradation, and DNA translocation, among others^1^. Various aspects of the operation of ring-shaped motors such as force generation, substrate engagement, mechano-chemical conversion, and inter-subunit coordination have been described^2–9^. In addition, recent high-resolution structures of ring-shaped ATPases (in particular helicases and polypeptide translocases) have revealed lock-washer architectures^10–12^. Based on these structures, a hand-over-hand mechanism of translocation has been proposed, in which the crack in the ring precesses along the subunits, and the motor imposes its helical structure on the otherwise disordered substrate. The question then arises as to what is the mechanism of operation of ring ATPases that adopt lock-washer conformations but translocate structured substrates, such as double-stranded DNA (dsDNA)?

To tackle this question, we investigate how the structure and symmetry properties of the substrate determine the stepping mechanism of the DNA-packaging motor of bacteriophage ϕ29, which has recently been shown to adopt a lock-washer structure in which the individual subunits contact the phosphates of the DNA backbone^13^. This homopentameric motor displays a mechano-chemical cycle of bursts—during which 10 bp of dsDNA are internalized in the viral capsid—segregated from dwells—wherein the motor subunits exchange ADP for ATP in a sequential manner around the ring^3^. Moreover, each burst is made of four 2.5 bp steps (0.85 nm), indicating that one of the five subunits is special in that it does not perform a mechanical but a regulatory function^5,7^. This task requires the special subunit to contact a pair of phosphates in the strand that is being packaged in the 5′ to 3′ direction (the tracking strand) every pitch^4^. All of these properties are incompatible with the hand-over-hand mechanism proposed in the literature, where nucleotide exchange and hydrolysis are interlaced, every subunit is functionally identical, and translocation occurs in pairs of substrate monomers^10–12^. Hence, different mechanisms of translocation have been proposed for ϕ29; however, definitive evidence for any of them is missing^3,14^.

To gain insight into this mechanism, we monitored the operation of the ϕ29 packaging motor on double-stranded RNA (dsRNA) and DNA/RNA hybrid substrates, whose helical periods are shorter than canonical B-form DNA. Significantly, we find that the motor adapts its burst size to match the new substrate’s periodicity. However, we find that three of the individual steps within the modified burst retain their original size—indicating that these are fixed and determined only by the conformational changes of the subunits concomitant with ATP hydrolysis—whereas the fourth is reduced to match the pitch of the double helix. Finally, although the motor packages continuously when the tracking strand is DNA, packaging is interrupted by reverse-translocation events composed of consecutive slips of burst-sized steps when it is RNA. Based on these observations, we propose a model for the packaging mechanism in which the ring cracks open, progressively adopting a lock-washer conformation during the ATP loading dwell, and successively returns to a planar structure upon sequential hydrolysis during the translocation burst.

## Results and discussion

### The motor can translocate A-form substrates

**Fig. 1:**
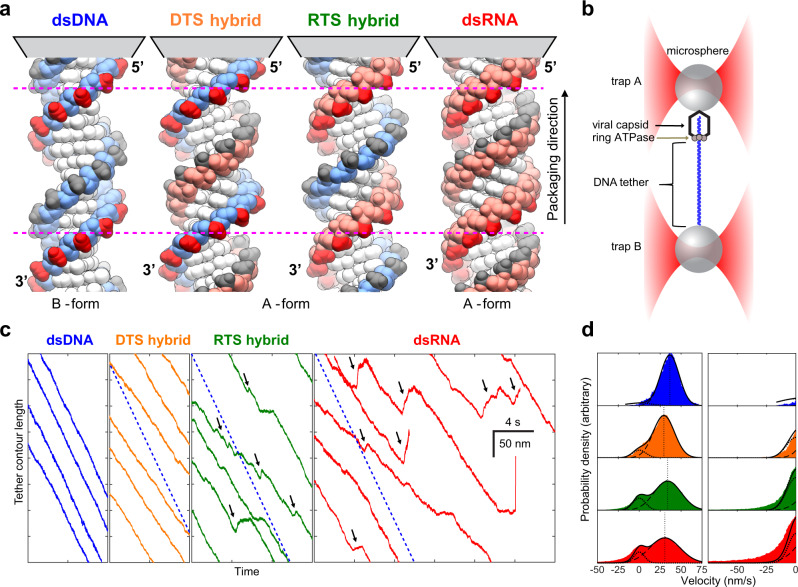
Packaging of alternative substrates. **a** The structure of the four substrates is shown, with DNA and RNA strands in light blue and light red, respectively. The strand being packaged from 5′ to 3′ is designated the tracking strand and its phosphates are colored dark red. Note the differing orientation of the phosphates for DNA and RNA strands, and the differing helical pitches (Table 1, red dotted lines span one pitch of dsDNA). **b** Single packaging complexes are held between two microspheres using a dual-trap optical tweezer, whereas the tether length is monitored over time. **c** Representative packaging trajectories for the four substrates are shown at 250 Hz. Dashed blue lines represent the velocity of packaging on dsDNA substrate. Reverse-translocation dynamics are observed on the RTS hybrid and dsRNA (black arrows). **d** Velocity distributions for the different substrates are curve fit to the sum of two gaussians (dark line), one with zero mean (curved dotted line) and one with positive mean (curved dashed line), which represent pausing and packaging, respectively. Vertical straight dotted lines mark the mean of the corresponding positive velocity gaussian fit. The right panel details negative velocities, which correspond to reverse-translocation dynamics. Note the increased probability of these events on dsRNA compared to the RTS hybrid. For all panels, [ATP] is 0.25 mM and opposing force is 7–12 pN.

The natural substrate of the ϕ29 packaging motor is dsDNA, which adopts the canonical B-form under physiological conditions. In contrast, dsRNA and DNA/RNA hybrids adopt exclusively the A-form, displaying shorter helical pitches, while the main features of their solvent-exposed phosphodiester backbones and base-pair stacking cores remain essentially the same^15–18^ (Fig. 1a and Table 1). We challenged the ϕ29 packaging motor with these alternative structures and monitored its translocation at the single-molecule level using high-resolution optical tweezers. We designate the DNA/RNA hybrids either as a DNA-tracking strand (DTS) hybrid or RNA-tracking strand (RTS) hybrid, depending on the chemical identity of the strand being packaged in the 5′ to 3′ direction (Fig. 1a).

**Table 1:** Structural and packaging features of the four different substrates.

Structural/Packaging data	dsDNA	DTS hybrid	RTS hybrid	dsRNA
Periodicity (bp/turn)	10.4	10.8^a^	10.8^a^	11
Helical pitch (nm/turn)	3.52	3.11^a^	3.11^a^	3.02
Burst size (nm)	3.39 ± 0.03	3.09 ± 0.02	3.02 ± 0.02	2.70 ± 0.02
Dwell time (ms)	86 ± 0.8	105 ± 0.7	85 ± 0.5	83 ± 0.7
*N* traces	90	69	65	67
Forward pause-free velocity (nm/s)	36.6 ± 0.5	29.4 ± 0.2	33.9 ± 0.3	30.6 ± 0.3

^a^Average taken from four different DNA/RNA hybrid structures (see “Methods”).

Individual packaging complexes were tethered between two microspheres in a dual-trap optical tweezers instrument^3^ (Fig. 1b) and the tether’s end-to-end distance was monitored over time, while the motor packaged one of the three A-form substrates in the presence of ATP. Figure 1c depicts the packaging trajectories obtained over these alternative substrates compared with those over dsDNA at saturating [ATP] (0.25 mM ~8 × *K*_M_*)* and 7–12 pN of opposing force^2^. The motor packages all A-form substrates, which rules out a recent model of motor operation that proposes the translocation mechanism involves a B-form to A-form transition of the substrate induced by dehydration and driven by ATP hydrolysis^14,19^, as dsRNA cannot adopt the B-form.

The motor packages the hybrids and dsRNA slower than dsDNA, with average packaging velocities of 36.6 ± 0.5 nm/s for dsDNA, 29.4 ± 0.2 nm/s for the DTS hybrid, 33.9 ± 0.3 nm/s for the RTS hybrid, and 30.6 ± 0.3 nm/s for dsRNA (all values mean ± SEM) (Fig. 1c, d and Table 1). Significantly, when packaging the RTS hybrid and dsRNA, the motor displays reverse-translocation events during which the tether length increases with a finite slope for a period of time until eventually it resumes packaging (Fig. 1c, black arrows). In contrast, when packaging dsDNA and the DTS hybrid, the motor does not exhibit these events, suggesting that the cause of the reverse-translocation events is weaker motor–substrate interactions when the tracking strand is RNA. Figure 1d displays the velocity distributions obtained for the different substrates, wherein the reverse-translocation dynamics manifest as long tails of negative velocities in the distribution.

### The motor burst adapts to the helical pitch

**Fig. 2:**
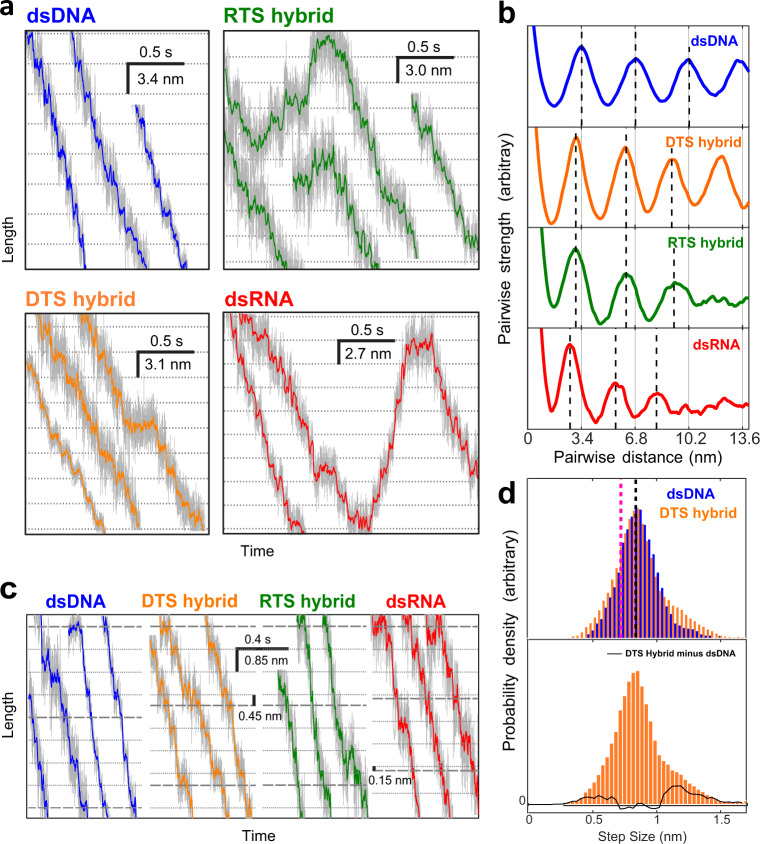
Burst and step sizes for different substrates. **a** Representative packaging traces at low force (7–12 pN) are shown for the four substrates at 2.5 kHz (gray) and are filtered to 100 Hz (colored). On A-form substrates, the dwell-burst coordination of the motor is retained but with a changed burst size. The dashed lines are spaced to match the mean burst size of the substrates. **b** Pairwise distance analysis of the trajectories confirms a decrease of the burst size for the three A-form substrates. The dashed lines are evenly spaced in multiples of the mean burst size. **c** Representative traces at high force (35–40 pN) resolves motor steps. For dsDNA, steps of 0.85 nm are observed; however, for A-form substrates, smaller steps are also seen every four steps. **d** Step-size distributions from packaging traces against high-force. Top: the step-size distribution of dsDNA and DTS hybrid peak at 0.85 nm (black dashed line). The striking similarity between maxima supports the interpretation that the burst adapts to the decreased helical pitch by modifying one of the four steps over the alternative model where the four steps are proportionally reduced. Would the latter be the case, the DTS hybrid step distribution would be expected to peak at 0.72 nm (magenta dashed line). Bottom: by taking the difference between the dsDNA and the DTS hybrid step-size distributions (black solid line), we observe two populations of steps whose means are 0.51 and 1.36 nm, respectively. These sizes are consistent with the size of the expected fourth reduced step and its combination with one regular 0.85 step, as if the two steps were combined during stepfinding. For all panels, [ATP] is 0.25 mM.

To understand how the motor processes these different substrates, we analyzed packaging trajectories at high resolution. With all three A-form substrates, the motor conserves its dwell-burst operational scheme (Fig. 2a). The dwell times for the RTS hybrid and dsRNA are similar to those of dsDNA (Table 1). Only the DTS hybrid values show a significant difference—we do not have an explanation for this behavior. Moreover, the dwell times are γ-distributed on all four substrates, just as it was previously described for dsDNA^3,5^, an indication that nucleotide exchange of the motor subunits is essentially unaltered by the A-form structures (Supplementary Fig. 1 and Supplementary Discussion). Importantly, however, we find that the bursts are smaller for all three A-form substrates (Fig. 2a). The pairwise distance distribution analysis of high-resolution packaging trajectories (Fig. 2b) reveals that the burst size for the A-form substrates is reduced to 3.1 ± 0.02, 3.0 ± 0.02, and 2.7 ± 0.02 nm for the hybrids and dsRNA, respectively, and to 3.4 ± 0.03 nm for dsDNA (all values mean ± SEM). Significantly, these burst sizes closely match the corresponding helical pitches of the substrates (Table 1).

To determine the size of the steps that compose the reduced bursts, we obtained packaging trajectories under 30–35 pN of opposing force and performed a stepfinding analysis (see “Methods”). On dsDNA, as previously reported, the burst is made up of four steps of 0.85 nm^3^. On the DTS hybrid, we find that each burst is instead composed of three 0.85 nm steps and one smaller step of 0.45 nm (Fig. 2c, d). In addition, we also observe 0.85 nm steps on the RTS hybrid and dsRNA, but slipping frequency and resolution limitations in these substrates prevent the quantitative determination of the smaller steps (Supplementary Discussion). However, as the total size of the burst on the RTS hybrid and dsRNA are 3.0 and 2.7 nm, respectively, we determine that the shortened step for the former substrate is 0.45 nm and 0.15 nm for the latter (Fig. 2c).

These results demonstrate that (i) the inherent step size of the motor subunits is most likely determined by the conformational change that accompanies their power stroke during phosphate release and (ii) to successfully package a reduced helical pitch substrate, the motor decreases the size of one power stroke during each burst, possibly due to the earlier encounter of the last subunit with the backbone phosphate one pitch away in the helix^4^. As the cryogenic electron microscopy (cryo-EM) lock-washer structure of this motor shows that it spans one pitch of a dsDNA helix^13^, our results suggest that the motor adapts to the A-form substrates, opening less on them by distorting one of the inter-subunit interfaces.

### Burst-size slipping on RTSs

**Fig. 3:**
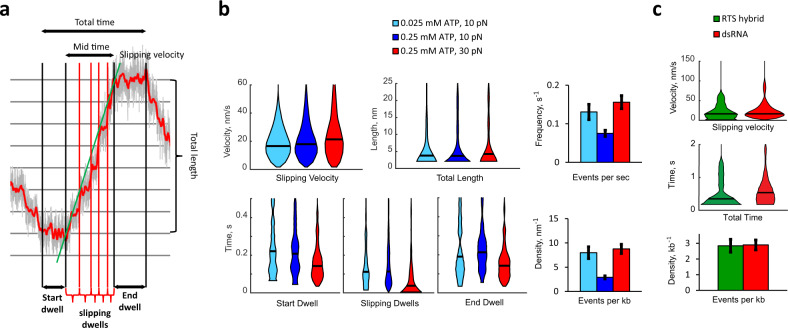
Reverse-translocation dynamics. **a** An individual reverse-translocation event is divided into three sections to differentiate the first and last dwell from the dwells during burst-sized slipping (slipping dwells). **b** Violin plots and bar graphs of select aspects of the reverse-translocation events on dsRNA under different [ATP] and opposing force are shown. Data are from *n* = 57, 85, and 83 reverse-translocation events for 0.025 mM [ATP] 10 pN, 0.25 mM [ATP] 10 pN, and 0.25 mM [ATP] 30 pN, respectively. **c** Violin plots and bar graphs showing the differences of select statistics when the substrate is the RTS hybrid compared to dsRNA. Data are from *n* = 21, 85 reverse-translocation events for RTS hybrid and dsRNA, respectively. As the total length is equal to the slipping velocity times, the mid-time, the trend in total length is identical to that of the mid-time graph, as the slipping velocities for RTS hybrid and dsRNA are the same. [ATP] is 0.25 mM and the opposing force is 10 pN. For **b** and **c**, horizontal black lines on the violins are the median of the corresponding distribution and errors on bar graphs are SEM.

Surprisingly, the reverse-translocation events observed only on the RTS hybrid and dsRNA occur in increments whose sizes coincide with the magnitude of the burst on the corresponding substrate (Figs. 2a and 3a, and Supplementary Fig. 2). Therefore, we refer to them as burst-sized slips. The appearance of burst-sized slipping only when with RTS hybrid and dsRNA suggests that critical interactions between subunits in the motor and the DTS confer a stable grip during packaging, and that these can be sporadically lost when this strand is RNA. The brief periodic dwells separated by sudden slips presumably reflect the motor weakly gripping the substrate through the same phosphate contacts it makes during a normal packaging dwell^4^ and releasing it to grip it again one full turn away.

We propose that during the reverse-translocation events, the motor transitions into a packaging-incompetent (PI) state that has reduced grip for the substrate. To analyze burst-sized slipping, each reverse-translocation event is divided into three sections: (i) the start dwell, where the motor transitions into the PI state before burst-sized slipping starts; (ii) the slipping dwells, which separate the burst-sized increases in tether length; and (iii) the end dwell, where the burst-sized slipping ends and the motor transitions back to the packaging-competent (PC) state (Fig. 3a and Supplementary Fig. 2).

The length slipped during an event does not increase with limiting [ATP] (10–25 μM) (Fig. 3b) and, as the length per slip is constant (one burst), we must conclude that the number of slips also does not increase with limiting [ATP], suggesting that nucleotide exchange is halted during the course of a slipping event. Limiting [ATP] also increases the frequency of reverse-translocation events, indicating that the transition from the PC to the PI state takes place when the motor is not fully saturated with ATP (Fig. 3b). On the other hand, the duration of the start dwell and the total extension of the reverse-translocation event are independent of [ATP], implying that, although in the PI state, the motor is unable to exchange ADP for ATP (Fig. 3b). Moreover, these observations suggest that during a regular packaging dwell, a kinetic competition ensues between the completion of ATP exchange in the PC state and the attainment of the PI state (Supplementary Fig. 3).

As the slipping dwells are much shorter than the start dwell, we conclude that the motor is in a different state during slipping than when it first entered the PI state during the start dwell. Therefore, at some point during the start dwell, the motor must make a transition from a PI-gripping state to a PI-slipping state (Supplementary Fig. 3). Finally, we note that the end dwell is lengthened at limiting [ATP] (Fig. 3b), indicating that the motor, presumably not fully saturated with ATP, transits from the PI-gripping state to the PC state where it can restart its halted nucleotide exchange and resume packaging (Supplementary Fig. 3).

Taken together, all the above observations lead us to conclude that in the PI state the motor–substrate interaction has been compromised resulting in the halting of nucleotide exchange and the hydrolysis cascade^7^. Although in the PI-slipping state, the motor, in its ATP-unsaturated state, is unable to stably hold onto the RTS, eventually slipping a full turn of the helix successively. The inability of the motor to make the proper phosphate contacts with the tracking strand of the A-form substrates explains the interruption of the mechano-chemical events of the motor’s cycle. In addition, previous studies have shown that this motor and similar viral dsDNA translocases display a decreasing grip for the substrate when subunits in the ring are in the ATP, ADP, and apo forms, respectively^2,20^. Hence, ATP saturation of the motor confers strong grip for its substrate via the tracking strand. Furthermore, the length of time the motor spends in a reverse-translocation event is two times longer on a dsRNA substrate than on an RTS hybrid (Fig. 3c). This observation indicates that the rate at which the motor regains stable grip is sensitive to the structural details of the tracking strand, presumably the helical pitch, which is shortest for dsRNA, and/or other structural differences between the tracking strands (Fig. 1a and Table 1).

### A helical inchworm model of translocation

**Fig. 4:**
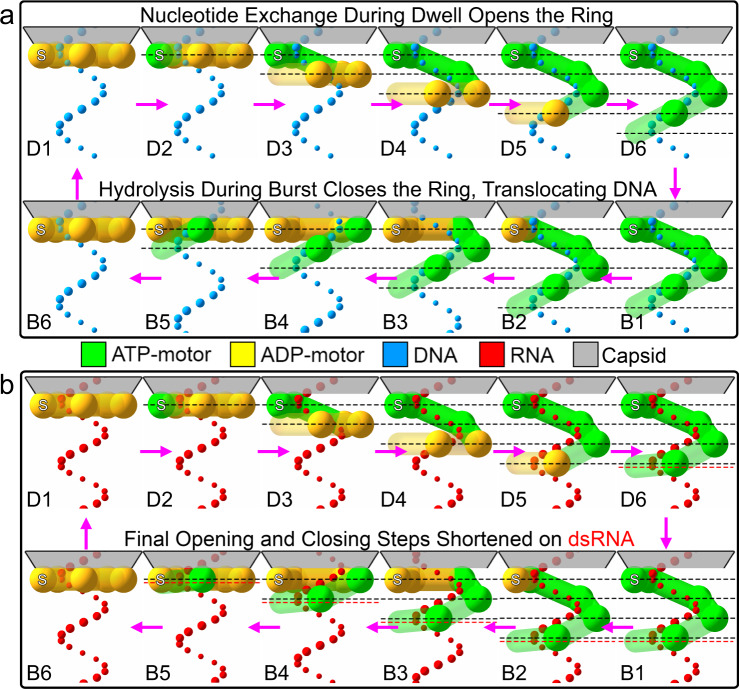
Translocation mechanism for the ϕ29 DNA-packaging motor. The mechanism of the motor packaging (**a**) dsDNA and (**b**) dsRNA is shown. For each state, the ring ATPase complex is presented from the side, with each subunit represented as a cylinder with a sphere at its hinge and colored by nucleotide state. The capsid is above in gray and the special subunit is on the left, marked with an S. Tracking strand phosphates are depicted as colored spheres. The states in the dwell are labeled as D1–D6 and the states in the burst are labeled B1–B6. D6 and B1 are the same state, as well as D1 and B6, repeated only for horizontal comparison. Dashed lines with spacing 0.85 nm show the extent of ring opening. One phosphate is colored magenta during the burst to track translocation. During the dwell, ADP exchanges for ATP, which opens the ring. During the burst, ATP hydrolysis drives DNA translocation. It is noteworthy that the first exchange and hydrolysis is in the special subunit, which causes no ring opening or substrate translocation. On dsRNA, the mechanism is identical, except for the final opening and closing steps, which are shorter and are denoted by the red dashed lines. See also Supplementary Movie 1 and 2.

The reduced grip of the motor for substrates harboring an RTS of decreasing helical pitch suggests that during translocation the motor adopts a lock-washer conformation in which its subunit interact optimally with the tracking strand of dsDNA. Moreover, the correlation between increased grip and ATP saturation (Fig. 3b) suggests that the interaction between the motor and the tracking strand is progressively established as the subunits in the motor sequentially bind ATP during the dwell. These observations are not easily rationalized by a hand-over-hand model in which the motor maintains its helical symmetry and its degree of ATP saturation before every step, which would result in a constant grip throughout the cycle. Likewise, in a model in which the lock-washer structure is preserved during the translocation burst, while steric paddles translocate the substrate^3^, the motor would lose its register with the tracking strand after just one step, abolishing its strong grip for the substrate. On the other hand, if during the burst, the motor gradually converts from the lock-washer structure to a planar form as it translocates the substrate, it could partially preserve its grip on it throughout the process. Indeed, evidence that the motor can also adopt a planar structure has been recently found in X-ray and single-particle cryo-EM structures of the packaging ATPase of the closely related phage asccphi28 obtained without DNA in the apo- and ADP-bound forms^13^. Based on these observations, we propose a translocation model in which, during its mechano-chemical cycle, the motor converts between a planar and a lock-washer structure. A similar model has been proposed previously for this motor, although without any experimental evidence to support or rule it out against the steric paddle model^3^. In this model, at the beginning of the dwell, the motor is in a planar structure that displays its weakest grip for the substrate, because only the special subunit, numbered here as the first in the ring, is in direct contact with the tracking strand^4,7,21^. As exchange of ADP for ATP proceeds, hinge-like displacements between the first and second subunits, the second and third, and so on ensue, breaking the interface between the first and fifth subunits, resulting in a lock-washer structure (Fig. 4a). At the end of this process, each subunit now has established electrostatic interactions with phosphates in the tracking strand, which confer the motor its strong grip when fully loaded with ATP. Although it has been previously established that the larger the amount of [ADP] in the medium, the weaker the grip of the motor for its substrate^2^, this model predicts that the loss of grip is directly proportional to the number of ATP molecules bound to the motor at any given time. Such an assumption in our model awaits direct experimental confirmation. These successive conformational changes are fixed and optimized to match the pitch of dsDNA. To explain how the motor matches the shorter pitch of the A-form helices, we propose that with these substrates, the displacement of the fifth subunit is partially interrupted by its earlier encounter with its corresponding phosphate in the tracking strand (Fig. 4b).

During the burst, each subunit, following hydrolysis and phosphate release^2^, propels the substrate, sequentially returning to their conformation prior to ATP binding, progressively restoring the planar structure of the motor, while translocating the substrate. The order in which the subunits hydrolyze their nucleotide during ring closing must be the same as that in which they exchanged nucleotide during ring opening, as this scheme preserves the greatest number of motor–substrate contacts (Fig. 4a) and is consistent with the highly coordinated mechano-chemical cycle previously proposed for this motor^5^. Each hydrolysis/phosphate-release event serves as the signal for the next subunit to hydrolyze its ATP, resulting in the four sequential steps that compose the burst. This model provides a straightforward explanation for the division of labor among the subunits, previously described for this motor. Specifically, the special subunit, proximal to the capsid and with a broken inter-subunit interface, cannot undergo a power stroke; its ATP hydrolysis instead signals the beginning of the burst^5,7^ (see Supplementary Discussion and Fig. 3a). By the time the distal (fifth) subunit executes its power stroke, the motor has regained its planar structure and the phosphate in the tracking strand that was one full pitch down at the beginning of the cycle has been brought in register to contact the special subunit at the cycle’s end, most likely with the aid of some small substrate rotation^22^. With the dsDNA substrate, the burst is made up of four identical steps of 0.85 nm each (2.5 bp of dsDNA). With the A-form substrates, the last step of the burst is shorter due to the interrupted hinge movement of the fifth subunit during the dwell. The way the two ends of the open ring walk down the helical substrate is similar to the manner in which the two ends of an inchworm move as it walks; hence, we name this model the helical inchworm (Fig. 4).

Hand-over-hand mechanisms of translocation have been recently proposed for ring ATPase motors that adopt a lock-washer architecture and operate on disordered substrates. The hallmark of these models of operation is the functional requirement that, after one ATP hydrolysis-powered step, the motor must perform nucleotide exchange in order to return to the pre-translocation state^10–12^. Throughout the whole cycle, the helical structure of the motor is maintained. Here we studied the dsDNA-packaging motor of bacteriophage ϕ29 as a model of a ring-shaped translocase that operates on a substrate possessing a pre-existing structure. By using alternative double-helical structures, we have revealed that the motor is capable of adapting itself to the periodicity of the substrate and of maintaining the inter-subunit coordination throughout its mechano-chemical cycle. We propose a model of translocation in which the ring converts between a lock washer and a planar structure during its mechano-chemical cycle, in a way that maximizes its contacts with the spiral shape of the substrate. Future work is needed to obtain definitive evidence of the proposed mechanism, either by capturing some intermediate states of the motor via cryo-electron microscopy or by following the ring opening via changes in fluorescence resonance energy transfer, whereas DNA packaging is co-temporally monitored in an optical tweezers channel. These efforts are currently underway. It is noteworthy that the helical inchworm model explains the high forces (60 pN) against which the motor can package the viral genome and its ability to resist the internal pressure (>30 atm) that builds up in the capsid during packaging^22–24^, for which there has been, until now, no rationalization. Similarly, the cycling between a planar and a helical configuration implied by the helical inchworm mechanism is the most parsimonious model that is consistent with the well-established segregation between a nucleotide exchange phase (where the motor transits from planar to helical) and a nucleotide hydrolysis phase (where the motor converts from helical to planar). The flexibility of the motor to package substrates of different helical pitches may reflect the need to accommodate its operation to the natural sequence-dependent structural variability of dsDNA (23). This natural adaptability may also explain why this motor can package DNA against external forces as high as 60 pN, wherein the double helix is expected to stretch by about 5%. Interestingly, the ϕ29 motor has been shown to also package stretches of single-stranded DNA (ssDNA) (4). It is possible that in this case the motor, while maintaining its translocation mechanism, imparts its helical symmetry on ssDNA, as has been shown for disordered substrate translocases. We speculate that the helical inchworm model proposed here may underlie the operation of other ring-shaped dsDNA translocases, as well as that of others that operate on disordered substrates.

## Methods

### Modified substrates

Supplementary Fig. 4a shows a schematic of the substrate geometry. Two complementary ssRNA transcripts of 4 and 3.8 kb were generated using MEGAscript T7 vitro transcription (Themo-fisher Scientific) and were purified using MEGAclear (Themo-fisher Scientific). The corresponding PCR templates were amplified from λ phage DNA (NEB), with ~50% GC content; the same sequence is used for the 4 kb dsDNA substrate. For the DTS hybrid, the 5′-end of the 4 kb transcript was biotin-labeled using Vaccinia Capping System (NEB) and 3-biotin-GTP (NEB), and was purified using MEGAclear. The complementary DNA was synthetized using Protoscript II reverse transcriptase (NEB) and an adequate standard primer (IDT). For the RTS hybrid, the reverse-transcription step is done using a 5′-biotinylated primer (IDT). For dsRNA, the two transcripts were annealed at 65 °C for 15 min and cooled at a rate of 5 °C every 10 min until 25 °C, in 15 mM PIPES pH 6.5, 10 mM EDTA. The 187 nt overhang was completed using Protoscript II reverse transcriptase. For pulling experiments, only the 5′-biotin-labeled 4 kb RNA transcript was used and for the corresponding reverse-transcriptase reaction the primer was 5′-digoxigenin labeled. The sequences of the used primers are available in Supplementary Table 1.

### Optical tweezers packaging experiments

A dual-trap optical tweezers instrument^3^ was used to carry out single-molecule packaging experiments using two polystyrene beads of 1 μm in diameter each. Trap stiffnesses were in the 0.4–0.7 pN/nm range. Raw data were acquired at 2.5 kHz. The instrument was controlled using custom software written in LabVIEW.

ϕ29 prohead-motor complex^3,4^ was made by incubating purified proheads with recombinant motor ATPase (gp16) for 5 min at room temperature, after which ATPγS was added to a final concentration of 50 μM and kept for 24 h at 4 °C.

Single packaging tethers were obtained following the in situ initiation protocol^3,4^ where one polystyrene bead was deposited with substrate, whereas the second bead was deposited with a prohead-motor-ATPγS complex. The first bead was a streptavidin-coated polystyrene microsphere and the second bead was a carboxyl-functionalized polystyrene microsphere (both Bangs Laboratories) conjugated with antibody against gp8, a viral capsid protein. In situ initiation of packaging occurs after few seconds of rubbing the two trapped beads in the presence of ATP. The packaging buffer (0.5× TMS) contained 25 mM Tris-HCl pH 7.8, 50 mM NaCl, 5 mM MgCl_2_ in 80% D_2_O, 20% H_2_O. [ATP] was 0.25 mM unless otherwise noted. Then, 100 mg/ml glucose oxidase, 20 mg/ml catalase, and 5 mg/ml dextrose (Sigma-Aldrich) were used as an oxygen-scavenging system to increase tether lifetime.

Single-molecule packaging trajectories were obtained by recording the decrease in tether extension (bead-to-bead distance) over time. Semi-passive mode was used to keep the force in a defined range, 7–12 pN (low force) or 30–35 pN (high force), for most experiments. Force-feedback mode was used to characterize the step-wise slipping events, to keep the force constant as the tether length increases.

Pulling experiments were performed over 4 kb fibers of the different polymers to characterize their mechanical properties^25–27^ in the packaging buffer. Force vs. extension pulling curves (Supplementary Fig. 4b) were fitted to the extensible worm-like chain model, to experimentally obtain the persistence length (50, 35, and 40 nm) and the stretch modulus (900, 700, and 500 pN) for dsDNA, DNA/RNA hybrid, and dsRNA, respectively. These parameters were used to convert bead extension to tether contour for packaging experiments.

### Data analysis

Raw packaging trajectories were converted to contour length using the extensible worm-like chain model^2,3,28^. Velocity distributions were obtained by binning velocity data obtained by filtering traces obtained at low force with a Savitsky–Golay differentiating filter of order 1, width 301 points (8.3 Hz). These parameters were chosen, because they smooth the dwell-burst cycle of the motor (~11 Hz) but retain enough definition to capture the start of burst-sized slipping events. The bin size was 0.7 nm/s and the distributions were fit to two Gaussians, one with a fixed mean of 0 nm/s.

Pairwise distributions were generated by taking the autocorrelation of traces obtained at low force filtered by a moving average filter of 41 points (61 Hz). Distributions of individual traces were graded by their periodicity, by integrating the power spectrum over the frequency range corresponding to ±10% of the value of the average distribution’s peak^3^ and the top 30% of distributions were selected for selective averaging. A plot containing an average of all the distributions has been included in Supplementary Fig. S5.

Stepfinding on low-force data was done using the Kalafut–Visscher stepfinding algorithm^29^, to find the dwell times and the step sizes. The sensitivity parameter for the algorithm was similar to that used in previous studies on this motor^3,5^ and the mean step size found by the algorithm matches the periodicity determined by pairwise distribution. Burst-sized slipping was analyzed by finding steps against the direction of packaging, considering runs of such steps as one burst-sized slipping event. Stepfinding on high-force dsDNA and DTS Hybrid data was done using a hidden Markov model stepfinding algorithm^30^ to find the step size. The two step-size distributions were aligned and the difference was taken to generate the differential step distribution in Fig. 2d.

All analysis was performed using custom software written in MATLAB.

### Reporting summary

Further information on research design is available in the Nature Research Reporting Summary linked to this article.

## Supplementary information

Supplementary Information

Description of Additional Supplementary Files

Supplementary Movie 1

Supplementary Movie 2

Reporting Summary

## Data Availability

The data that support the findings of this study are available from the corresponding author upon reasonable request.

## Code availability

The LabVIEW code that was used in this study is available at 10.5281/zenodo.4632595 and the MATLAB code that was used in this study is available at 10.5281/zenodo.3718873. Both are licensed under GNU GPLv3 and A.T. may be contacted for details upon reasonable request.
