# Supplementary material for: A DNA packaging motor inchworms along one strand allowing it to adapt to alternative double-helical structures
Source: Nat Commun. 2021 Jun 8;12:3439. doi: 10.1038/s41467-021-23725-5 (PMC8187434; doi:10.1038/s41467-021-23725-5)
Supplement: Supplementary file 1 — Supplementary Information [file 41467_2021_23725_MOESM1_ESM.pdf]

Supplementary Information

Supplementary Discussion

The mechano-chemistry of the motor is preserved with A-form substrates

It has been described that in the dwell-burst cycle of the packaging motor the dwell times τ follow a gamma distribution, indicative of multiple rate-limiting events. These events were identified as ADP release by each subunit, which occurs sequentially around the ring[3,5]. The dwell time distributions observed with the A-form substrates are also gamma-distributed with similar scale and shape parameters (Extended Data Fig. 1). However for the RTS hybrid and dsRNA, the apparent number of rate limiting event, using the estimator $n_{min} = \langle\tau\rangle^2/(\langle\tau^2\rangle - \langle\tau\rangle^2)$, is 4.67 and 4.68 respectively, marginally smaller than for dsDNA and the DTS hybrid, which are 5.05 and 5.04, respectively (Extended Data Fig. 1). Such a small change in n_{min} may reflect a slight decrease in the ADP release rate by one of the subunits, for example, that of the special subunit because of its modified interaction with the RNA tracking strand. These differences notwithstanding, the mechano-chemical scheme of the motor is preserved when packaging alternative substrates.

Differing resolution limits with substrates

In dual-trap optical tweezers, the signal-to-noise ratio (SNR) is determined by the size of the beads used in the experiment and the stiffness *k* of the tether held between them[29]. The size of the bead cannot go appreciably below 1 μm, the size used in these experiments, and therefore the resolution is proportional to *k*. Under high forces (>10 pN), the substrates are fully extended and *k* is given by the stretch modulus of the tether (pN) divided by its length (nm). The measured stretch moduli of the dsDNA, hybrid, and dsRNA polymers are 900, 700, and 500 pN, respectively (Extended Data Fig. 5b). Therefore, the SNR of the hybrid and dsRNA is 0.78 and 0.56 times that of dsDNA, which explains why the data on the alternative substrates is noiser than that of dsDNA, and why it makes the resolving of the small 0.45 nm (0.15 nm) correction step for the hybrid (dsRNA) more difficult.

ATP hydrolysis cascade initiation by the special subunit

The strict order in which the mechano-chemical processes occur in this motor is paramount to its coordination, which in the helical inchworm model proposed here can be accomplished through trans-acting residues for all steps except for the initiation of the ATP hydrolysis cascade after the last subunit binds ATP[7]. For this step, a signal must be sent somehow from the subunit distal to the capsid, across the broken interface, to the special subunit. We attribute this to stress being built up in the motor subunits as the motor opens along the helix. By the time the last subunit has bound ATP, this stress buildup is sufficient to trigger hydrolysis by the special subunit, thus initiating the translocation cascade. This model also explains the spontaneous triggering of the translocation burst observed under very low [ATP], where the motor has not yet fully exchanged ADP for ATP[7]. Presumably, the sub-critical stress buildup in the ring at partial saturation allows the special subunit to eventually overcome the activation barrier required for firing when the motor has at least 3 subunits bound with ATP, since we never observe spontaneous triggering that results in a single translocation step, which would occur if 2 subunits were bound to ATP.

Primer name	use	Sequence (5'to 3')
4kbfwd	dsDNA	DIG-AACGCCAAAAAATATATTTATCTGCTTGAT (DIG = 5'digoxigenin modification)
4kbrev	dsDNA	BIO-CTTAAAAGAGAGATCCTGTGTT (BIO = 5'biotin modification)
Tx1fwd	3.8 kb RNA transcript	TAATACGACTCACTATTAGTGATATTTTTAGGCTTATCTACC
Tx1rev	3.8 kb RNA transcript	mGmGGCTTAAAAGAGAGATCCTGTGTTG (m = 2'-OMe modification)
Tx2fwd	4 kb RNA transcript	mAmACGCCAAAAAATATATTTATCTGCTTGAT (m = 2'-OMe modification)
Tx2rev	4 kb RNA transcript	TAATACGACTCACTATAGGGCTTAAAAGAGAGATCCTGTGTT
DTS_RT	DTS hybrid reverse transcription	AACGCCAAAAAATATATTTATCTGCTTGAT
RTS_RT	RTS hybrid reverse transcription	BIO-GGGCTTAAAAGAGAGATCCTGTGTTG (BIO = 5'biotin modification)
dsRNA_RT	dsRNA reverse transcription	BIO-AACGCCAAAAAATATATTTATCTGCTTGAT (BIO = 5'biotin modification)

Supplementary Table 1|Primers used to generate modified substrates

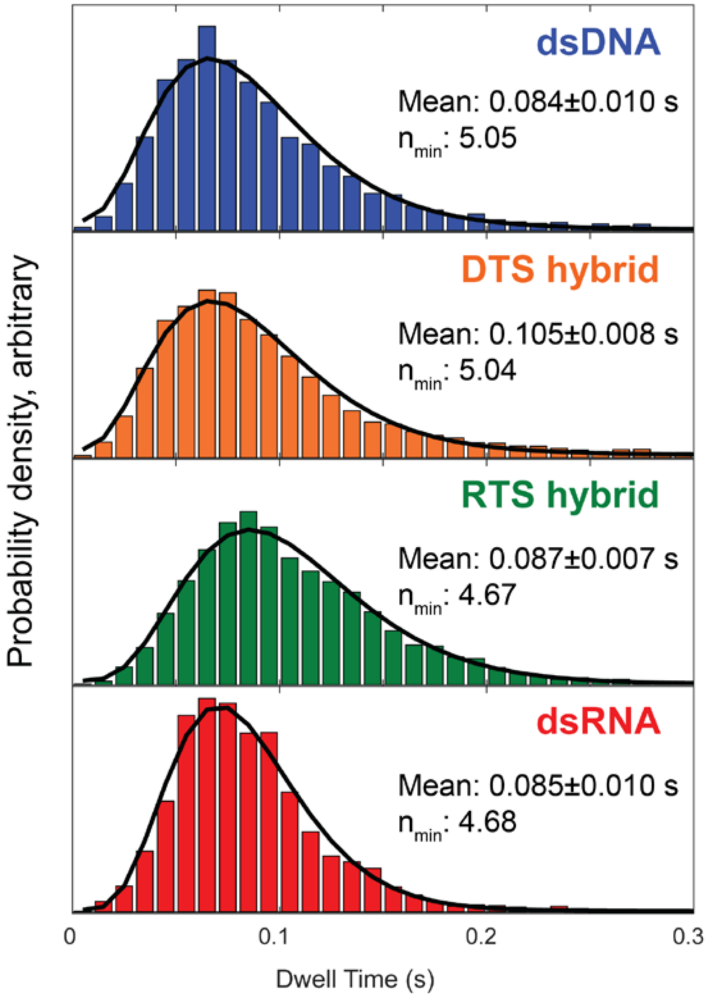

Supplementary Fig. 1|Dwell time distributions. The dwell times of the four substrates are obtained from stepfinding and are fit to a gamma distribution plus a single-exponential distribution via maximum likelihood estimation. The average of the gamma distribution (mean ± 95% C.I.) and the apparent number of rate limiting steps $n_{min} = <\tau>^2/(<\tau^2> - <\tau>^2)$ is noted. The single-exponential distribution accounts for the pausing seen in the alternative substrates, and represent events distinct from normal dwells.

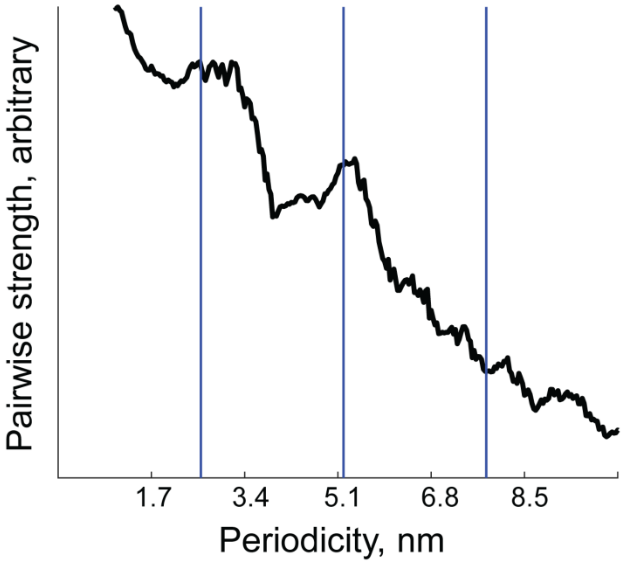

Supplementary Fig. 2|Pairwise distribution of reverse translocation events. A pairwise distribution is displayed of the dsRNA reverse translocation events to show their periodicity. Lines corresponding to multiples of the translocation burst size of dsRNA (blue) are overlaid.

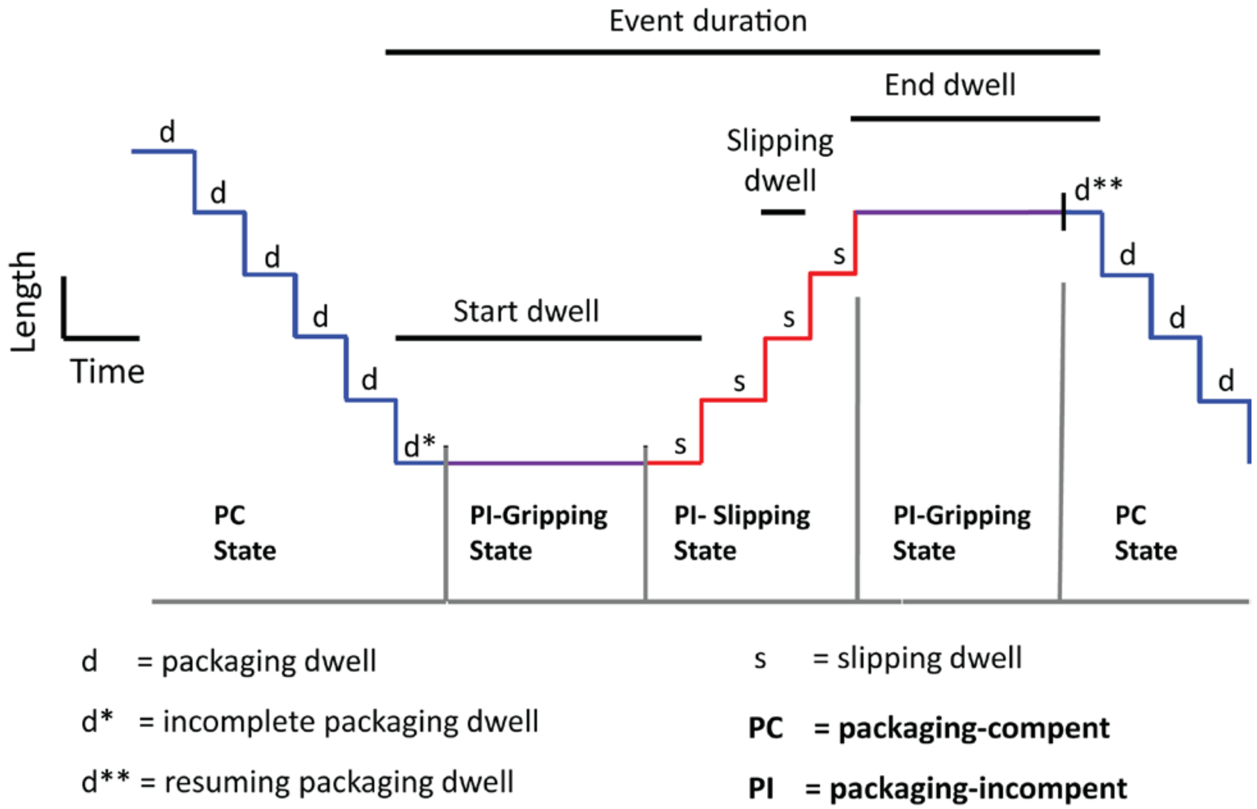

Supplementary Fig. 3 | Model of the reverse translocation events. A schematic packaging trajectory of a reverse translocation event is shown, with underlying states labeled. Blue, purple, and red lines correspond to the motor being in the packaging-competent (PC), packaging-incompetent (PI)-gripping, and PI-slipping states, respectively.

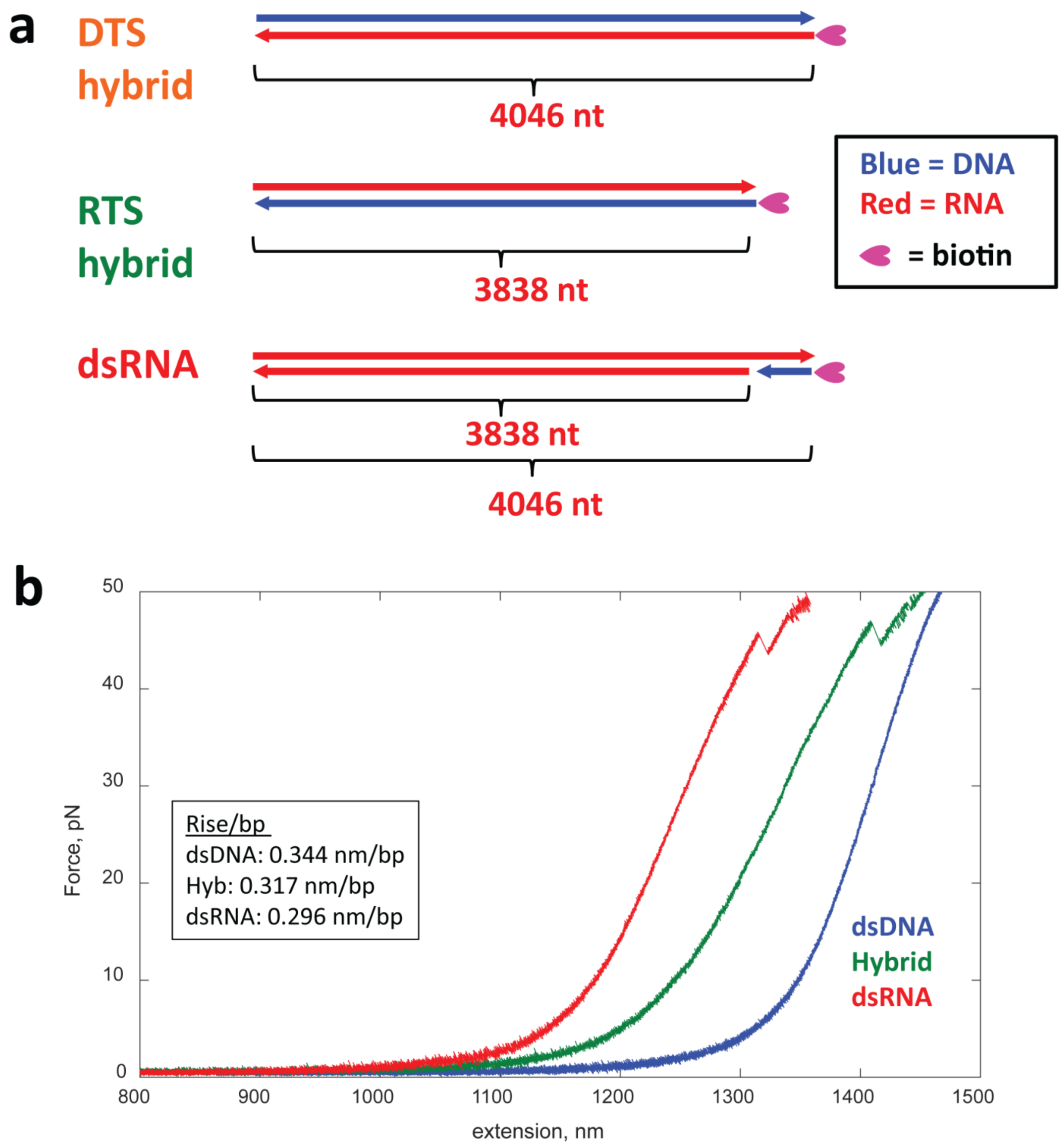

Supplementary Fig. 4 |Alternative A-form substrates construction and characterization. a. The construction of the alternative substrates is shown, where red solid lines represent RNA strands and blue lines represent DNA strands synthetized using reverse transcription (see methods). Arrow heads indicate 5'-to-3' direction. **b**. Force vs. Extension plot obtained from pulling experiments performed on 4 kb constructs of the different substrate polymers. DNA/RNA and dsRNA have a diminished contour length (left shift in the curves) compared to dsDNA, which means a smaller rise per base pair (see inset).

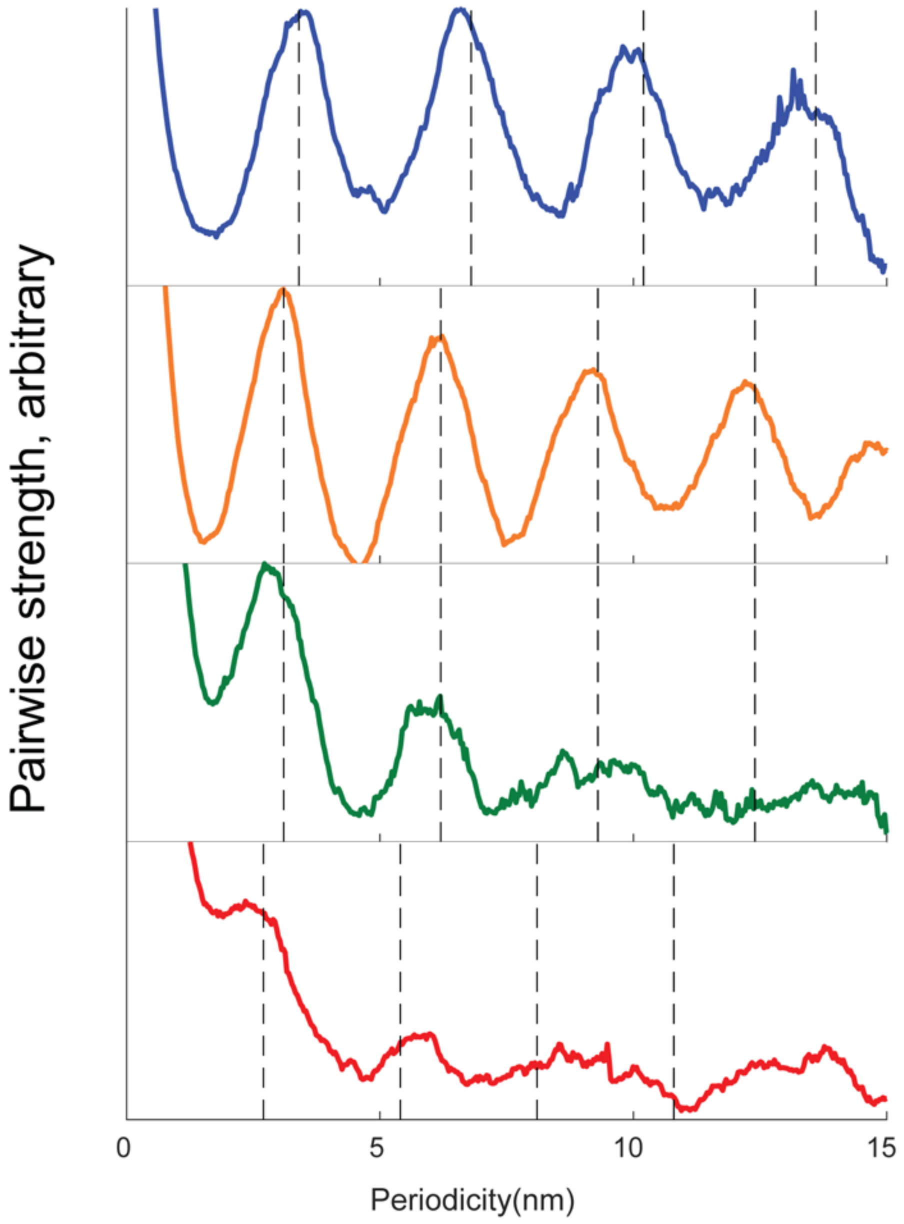

Supplementary Fig. 5 | Pairwise distance analysis of the full set of trajectories. The observed periodicities for all four substrates are preserved through the first and second peaks when considering all of the data, instead of selecting the top 30% of traces. The higher-order peaks are well defined for dsDNA and DTS Hybrid, however these are not well defined for the RTS and dsRNA substrates due to increased noise. The dashed lines are evenly spaced in multiples of the mean burst size (c.f. Fig. 2b).
