# Supplementary material for: A DNA packaging motor inchworms along one strand allowing it to adapt to alternative double-helical structures
Source: Nat Commun. 2021 Jun 8;12:3439. doi: 10.1038/s41467-021-23725-5 (PMC8187434; doi:10.1038/s41467-021-23725-5)
Supplement: Supplementary file 2 — Description of Additional Supplementary Files [file 41467_2021_23725_MOESM2_ESM.pdf]

Description of Additional Supplementary Files

File Name: Supplementary Movie 1
Description: The helical inchworm mechanism. For legend, refer to Figure 4.

File Name: Supplementary Movie 2
Description: Animation of the ϕ29 DNA packaging mechanism, by Grace Hsu (Animation Lab, University of Utah)
