# Supplementary figures and images for: A DNA packaging motor inchworms along one strand allowing it to adapt to alternative double-helical structures
Source: Nat Commun. 2021 Jun 8;12:3439. doi: 10.1038/s41467-021-23725-5 (PMC8187434; doi:10.1038/s41467-021-23725-5)

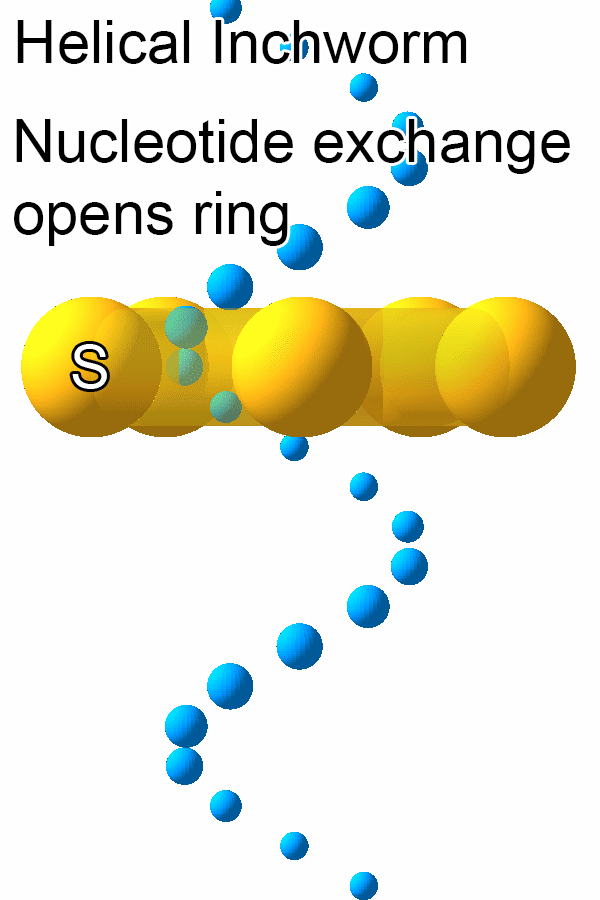

Supplement: Supplementary file 3 — Supplementary Movie 1 [file 41467_2021_23725_MOESM3_ESM.gif]
